# Examination of the “susceptibility gap” in the treatment of canine heartworm infection

**DOI:** 10.1186/s13071-017-2433-9

**Published:** 2017-11-09

**Authors:** Dwight D. Bowman, Jason Drake

**Affiliations:** 1 Department of Microbiology and Immunology, College of Veterinary Medicine, Cornell University, 930 Campus Road, Ithaca, NY 14853 USA; 2 ELANCO, Greenfield, IN USA

**Keywords:** Heartworm, Susceptibility gap, Treatment, Melarsomine, Macrocyclic lactone, Efficacy

## Abstract

**Background:**

The “susceptibility gap” in a dog diagnosed with adult heartworms has been defined as the period of time in which some *Dirofilaria immitis* stages are not susceptible to treatment with either macrocyclic lactones or melarsomine dihydrochloride. This was previously defined within the American Heartworm Society guidelines as a period of about 3 months “as per product labels.” It can be postulated, however, that a susceptibility gap does not exist with the combination of continued macrocyclic lactone therapy coupled with a three-dose melarsomine dihydrochloride protocol where the first intramuscular treatment is near the time of first diagnosis.

**Discussion:**

Melarsomine dihydrochloride was originally also investigated as a “preventive” as well as a treatment for adult heartworm infection where it would be given to dogs by intramuscular injection every 4 months; therefore, there was early interest in its ability to kill younger worms. A single intramuscular injection of 2.5 mg melarsomine dihydrochloride/kg has an efficacy of 82.1% against 4-month-old worms. When it was given to dogs with older heartworms, 7 and 12 months of age, a single injection was only 55.6% and 51.7% effective, respectively. Thiacetarsamide has been shown to be 99.7% effective against 2-month-old heartworms and other work has shown that melarsomine dihydrochloride is 100% efficacious against these younger forms. With the development and US Food and Drug Administration (FDA) approval of spinosad + milbemycin oxime (Trifexis®, Elanco), milbemycin oxime + praziquantel (Interceptor® Plus, Novartis, now Elanco), and milbemycin oxime + lufenuron + praziquantel (Sentinel® Spectrum®, Novartis, now Virbac), it was shown that repeated treatments of dogs with milbemycin oxime also has efficacy against 3-month-old heartworms. Thus, no improvement in efficacy is expected with a delay in initiating therapy with both melarsomine dihydrochloride and macrocyclic lactones, even with the presence of younger heartworms. Starting treatment at diagnosis appears to be acceptable for maximal heartworm clearance based on published data. Delaying treatment has the disadvantage of allowing disease progression and continued heartworm growth.

**Conclusions:**

The collective data that has been reviewed indicates that continued macrocyclic lactone administration with two additional injections of melarsomine dihydrochloride a month later will protect dogs against all heartworm stages, including those heartworms 2 months of age or younger at diagnosis, when both treatments are started upon diagnosis.

## Background

This paper examines the “susceptibility gap” in heartworm therapy. The “susceptibility gap” has been defined as the time between when a diagnosis of an adult heartworm infection is made and when any heartworms in the dogs are too old or too young to be susceptible to treatment with either a macrocyclic lactone or melarsomine dihydrochloride in the form of RM 340 (Rhône Mérieux) or Immiticide® (Merial). This paper examines the research publications related to the “gap” and suggests based on this review of the data that starting the treatment of dogs with both macrocyclic lactones and melarsomine dihydrochloride very near the time of diagnosis will cover all stages better than waiting 1 to 3 months to initiate melarsomine dihydrochloride administration. Overall, this paper shows that beginning concomitant treatment of a dog with a heartworm infection with a macrocyclic lactone and melarsomine dihydrochloride nearest to the time of diagnosis removes heartworms while they are most susceptible to treatment with both chemical classes and while they are likely smaller in size relative to starting treatment after a 1- to 3-month delay. This is because of the rapid growth of heartworms that occurs within a dog from 3 to 9 months post infection.

## Discussion

### The “susceptibility gap”

The “susceptibility gap” has been defined by the 2014 American Heartworm Society (AHS) Guidelines as being a period of about 2 to 3 months based upon the efficacies of the products “as per product labels” [1]. The current AHS recommendations are that upon diagnosis one should wait “2 to 3 months prior to administering melarsomine. This will reduce new infections, eliminate existing susceptible larvae, and allow older heartworms (between 2 and 4 months of age) to mature to a point where they would be more susceptible to melarsomine.” There are published data, however, that argue against this practice. First, a single intramuscular (IM) injection of 2.5 mg/kg melarsomine dihydrochloride killed 82.1% of heartworms 4 months post infection, but only 55.6% and 51.7% of heartworms, respectively, 7 or 12 months post infection [2]. It has also been shown that two IM treatments with melarsomine dihydrochloride given 24 h apart are 100% effective against 2-month-old heartworms [3]. Also, as will be discussed below, macrocyclic lactones are much more efficacious against growing heartworms than suggested “per product labels.” This paper discusses the “susceptibility gap” and questions the evidence for waiting 2 to 3 months to allow worms to mature to the point where they are susceptible to the “adulticide” effects of melarsomine dihydrochloride.

### Development of adulticide therapy

*Dirofilaria immitis*, the canine heartworm, remains one of the most pathogenic parasites of dogs [4]. Unfortunately, despite 30 years of readily available excellent, approved, and conveniently delivered preventives, the prevalence of infection has changed very little while the geographic spread has continued across North America [5–7]. Heartworm adulticide therapy has also evolved over the past several decades. Surgical removal of adult heartworms remains a necessity in the treatment of “caval syndrome” [8]; however, there have been major improvements in adulticide therapy. Through the 1980s, thiacetarsamide sodium (Caparsolate®, Abbot Laboratories) was the adulticide of choice for treatment of adult *D. immitis*. Thiacetarsamide sodium was administered intravenously, and dogs were typically hospitalized during initial treatment. In the 1990s, thiacetarsamide sodium therapy was replaced by melarsomine dihydrochloride (Immiticide®, Merial; now Boehringer-Ingelheim). This new treatment allowed for a simpler IM delivery. During the period of heartworm death after treatment, veterinarians typically avoid hospitalization of dogs undergoing therapy by requiring exercise restriction during this critically important aspect of patient care [9].

At the time of diagnosis, dogs are typically staged through observed clinical signs as being considered in one of four classes, 1 to 4 (Table 1). While melarsomine dihydrochloride therapy, with its improved efficacy and safety profile, is seen as a significant advancement compared with the use of thiacetarsamide sodium, many patients are not treated with melarsomine dihydrochloride for a variety of reasons. The main reasons given for avoiding adulticide treatment are commonly related to concerns about cost and safety [10, 11], and adverse events, associated with its administration and the subsequent heartworm death, do continue to be reported [4, 10, 12–19]. The melarsomine dihydrochloride protocol requires two to three administrations, and it is not always 100% effective. In 1992, a two-dose protocol (2.5 mg/kg, given IM, 24 h apart) demonstrated efficacies ranging from 95.8% to 100% of worms killed, with reports of 50% to 100% dogs being cleared of heartworms [2]. In order to reduce the risks of therapy to dogs with Class 3 heartworm infections that can be associated with the adulticidal effects of melarsomine dihydrochloride, a three-dose protocol was subsequently evaluated and recommended. In this three-dose method, a single dose administered and followed 30 days later by two doses, 24 h apart, has been shown to be 100% efficacious against male heartworms and 98% efficacious against female heartworms [2]. There are situations, however, when veterinarians are concerned about safety and post-treatment adverse reactions in a specific case or a pet owner objects to the cost of the treatment. Here, the attending veterinarian will often resort to utilization of a macrocyclic lactone regimen, sometimes in combination with additional medications.

**Table 1:** Classification of heartworm disease as defined in the Immiticide® (NADA 141-042) label

Class 1 – Asymptomatic to Mild	Patients in this category are characterized as having asymptomatic to mild heartworm disease. No radiographic signs or signs of anemia are evident. Patients with mild disease may have subjective signs such as a general loss of condition, fatigue on exercise, or occasional cough; however, no objective radiographic or other abnormal laboratory parameters will be present.
Class 2 – Moderate	Patients in this category are characterized as having moderate heartworm disease. Radiographic signs or signs of anemia [Packed Cell Volume (PCV) less than 30% but greater than 20%, or other hematologic parameters below normal] are evident. Mild proteinuria (2+) may be present. Radiographic signs may include right ventricular enlargement, slight pulmonary artery enlargement, or circumscribed perivascular densities plus mixed alveolar/interstitial lesions. Patients may be free of subjective clinical signs or may have a general loss of condition, fatigue on exercise, or occasional cough. If necessary, patients should be stabilized prior to treatment.
Class 3 – Severe	Patients in this category are characterized as having severe heartworm disease. These patients have a guarded prognosis. Subjective signs of disease may include cardiac cachexia (wasting), constant fatigue, persistent cough, dyspnea, or other signs associated with right heart failure such as ascites and/or jugular pulse. Radiographic signs may include right ventricular enlargement or right ventricular plus right atrial enlargement, severe pulmonary artery enlargement, circumscribed to chronic mixed patterns and diffuse patterns of pulmonary densities or radiographic signs of thromboembolism. Signs of significant anemia (PCV <20% or other hematologic abnormalities) may be present. Proteinuria (> 2+) may be present. Patients may have only moderate clinical signs and significant laboratory or radiographic alterations or they may have significant clinical signs with only moderate laboratory and radiographic signs and be categorized as Class 3. Patients in Class 3 should be stabilized prior to treatment and then administered the alternate dosing regime (See PRECAUTIONS and DOSAGE AND ADMINISTRATION).
Contraindications	
Class 4 – Very severe (caval syndrome)	Immiticide® is contraindicated n dogs with very severe (Class 4) heartworm disease. Patients in this category have caval syndrome (*D. immitis* present in the venae cavae and right atrium

The following parameters were used to classify the dogs in the clinical field trials for Immiticide®. Other parameters may be considered. As a general rule, conservative treatment should be employed because heartworm disease is serious and potentially fatal. If there is evidence of a high worm burden, patients should be categorized as Class 3

### Effects of preventives containing macrocyclic lactones against developing heartworms

Traditional heartworm prophylaxis, ie, killing <30 day-old larval worms, developed around the significant lethal effects that macrocyclic lactones and other anthelmintics, such as diethylcarbamazine citrate, have against the third-stage larvae and very young fourth-stage larvae of *D. immitis*. These tissue-stage larvae are eliminated when small, before they reach the pulmonary arteries where maturation and significant increase in body mass occurs. With the exception of the injectable, sustained-release formulation of moxidectin (ProHeart® 6, Fort Dodge, now Zoetis), all currently marketed products in the United States are designed for monthly administration targeting heartworms that have entered the dog in the last 30 days. Sustained-release, injectable moxidectin maintains blood/tissue levels that kill incoming larval stages less than 30 days old for a period of 6 months. Thus, all approved products have shown 100% efficacy against larvae less than 30 days of age. The susceptibility gap is defined with the very tight restrictions for “macrocyclic lactone susceptible (per product label)” and “melarsomine dihydrochlorides susceptible (per product label).” A great deal of work, however, has gone into the study of these molecules and their effects on different stages of heartworms, and there is very strong research support for the fact that there is no treatment gap if adulticide therapy is not delayed.

### Class 2 and class 3 heartworm treatment

In the case of Class 1, 2 and 3 heartworm-infected dogs that are going to receive the three-injection melarsomine dihydrochloride protocol (one injection followed a month later by an additional two melarsomine dihydrochloride injections administered 24 h apart), it is suggested by the AHS Guidelines that one wait 2 months prior to the first injection because of the “2-month susceptibility gap.” Thus, this discussion will begin by examining this protocol with the question being: Is there good reason to wait the 2 months? It is fully understood that a dog with a heartworm infection and associated heartworm disease may need to be stabilized before receiving melarsomine dihydrochloride treatment. It is common practice, however, to follow the guidelines and wait for almost all dogs before the first melarsomine dihydrochloride injection is administered, and this has become the basic all-encompassing recommendation in the 2014 AHS Guidelines [1].

Heartworms grow rapidly early in life, and this seems to argue against allowing them to get larger before they are killed. Males and females are <2 cm long when 60 days old, around 4 cm long when 90 days old, and at 120 days of age they are 7 cm (males) to 9 cm (females) in length. When they are 190 days old they reach lengths of 13 cm (males) to 20 cm (females) [20]. Thus, as heartworms mature, there is a greater mass (volume) of heartworms to die and decompose in the lungs of a dog after treatment, ie, if one waits 2 or 3 months, the bulk of any 90-day-old heartworms will increase four to five times, and the heartworm mass will increase even more substantially. An average 90-day-old female is 46 mm long and 0.18 mm in diameter and a 190-day-old average female is 205 mm long and 0.84 mm in diameter [21]. This is an approximate 100-fold increase in volume based on the formula for a simple cylinder, with the 90-day-old heartworms having a volume of 1.17 mm^3^ increasing to 113.6 mm^3^ in 190-day-old heartworms. Although nematodes have a voluminous pseudocoelomic cavity, there is also a massive increase in tissue, composed of genital, intestinal, muscle, and hypodermal elements, that markedly increases as these heartworms mature, and the external non-cellular collagenous cuticle (which degrades last as is apparent in histologic sections of dead adult heartworms) also thickens very markedly as the heartworms age. Thus, when one waits, the mass of the heartworms that will ultimately die in the lungs increases. Even waiting only a month will allow a 90-day-old heartworm to increase its volume by 17-fold as a 120-day-old heartworm (90 × 0.25 mm), ie, having a volume 17 times greater. If one is worried about 10 heartworms, then one will have an increase in the volume of the dead heartworms of anywhere from about 200 to 1000 times the volume of the 10 or so 90-day-old heartworms present when the heartworm infection is first diagnosed.

**Fig. 1:**
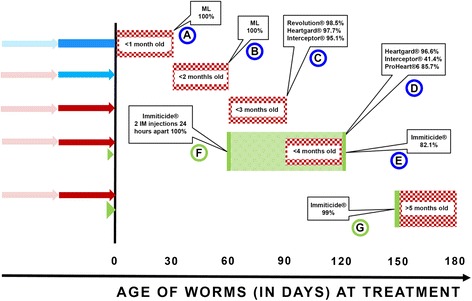
This illustration is predicated upon dogs beginning treatment with a macrocyclic lactone (ML) at the time of diagnosis and continuing the treatment for the next 6 months (or getting a second injection with the slow-release injectable product). The numbers along the horizontal line at the bottom indicate the age of worms present in a dog at the time of treatment (ie, diagnosis). The vertical line at the left represents the day the dog is diagnosed and treated. The bars and arrows to the left of the line represent the following: the blue bar represents the 100% efficacy as per the original CVM-FDA product approval; the blue arrow represents the 100% efficacy based upon published literature and so recognized by the American Heartworm Society; the red arrows represent continued treatments with MLs; the faint arrows represent that a next treatment will be administered. The small green arrowhead represents a single injection of melarsomine dihydrochloride at the time of diagnosis, and the larger green arrowhead represents the two injections that will be administered 24 h apart 30 days after the first injection. The red checkered boxes on the right of the vertical line show the ages of worms killed by repeated monthly treatment with macrocyclic lactones (MLs) where the callouts present the percent efficacies published for the MLs. The vertical green bars represent the treatment of dogs with melarsomine dihydrochloride (Immiticide®) against worms of given ages (see text for references). Beginning ML treatment on Day 0, the checkered box shows that if a dog is treated with an ML when the diagnosis is made that the treatment with a ML will be 100% efficacious against any larvae that are 30 days old or less. The second red checkered box shows that the repeated ML treatments will have 100% efficacy against worms less than 2 months old; thus, efficacy against worms less than 60 days of age with the two repeated ML treatments will be 100%. This red checkered box shows that efficacy against worms 90 days old or less with the tested products having efficacies of 95.1% to 98.5%. The red checkered box shows that the treatment with MLs at 120 days after dogs receive heartworm larvae will have efficacies of 41.4% to 96.8% against these worms that are 4 months old or less. A single injection of melarsomine dihydrochloride (Immiticide®) is 82.1% effective against 4-month-old adults (indicated by the green vertical bar at 120 days on the left end of the red box at the level of the smaller green arrowhead). The green vertical bar at 60 days represents what occurs if a dog is treated with two IM injections of melarsomine dihydrochloride when heartworms in the dogs are 2 months old, where efficacy was shown to be 100% (see text for reference), and the green checkered box between 60 and 120 days is suggesting that a single melarsomine dihydrochloride injection given to dogs with 120-day-old worms is likely to have efficacy against heartworms that are less than 4 months old as suggested by the 100% efficacy of the two IM treatments against 60-day-old heartworms. The last two melarsomine dihydrochloride injections a month after the singe injection should have an efficacy of 99% against adult worms that are 5 months old or older (indicated by the green vertical bar at 150 days)

All macrocyclic lactone heartworm preventives were originally approved by the FDA and Center for Veterinary Medicine (CVM) as having 100% efficacy against 30-day-old larvae. This is how the drugs are tested for approval. One gives a dog 30 to 100 (typically 50) infective third-stage larvae and then waits 30 days before the dogs are given a single treatment of the investigational product. (The exceptions here are the sustained-release products ProHeart® 6 and ProHeart® SR-12 (Fort Dodge, now Zoetis), where one first gives the dogs their injection of the investigational product, and then after either 180 or 365 days inoculate the dogs with infective larvae.). Thus, if a dog is given a single treatment with an approved dosage of a macrocyclic lactone heartworm preventive at the time of diagnosis, the product should have 100% efficacy against any heartworms that are ≤30 days of age that are present in the dog (Fig. 1, Bullet A). This is all that is covered by the “macrocyclic lactone susceptible (per product label)” on the AHS image of the susceptibility gap.

With regard to the first of the 3 remaining months within the gap, it seems there is excellent evidence that concern for another month can be eliminated. Based on a series of studies, the concept of “reach back” was developed where it was implied that multiple regular prophylactic doses of macrocyclic lactones had effects on growing heartworms of different ages beginning with worms 60 days post infection [21–25]. Based on this body of research evidence, the AHS put forth in the 2005 Guidelines the statement that “all macrocyclic lactones have a ‘reach-back’ of 2 months” and went on to suggest that the 2-month reach-back would provide protection even where poor compliance was an issue. Thus, if a dog is started on heartworm prevention at the time of diagnosis and is administered the preventive monthly thereafter, all heartworms less than 60 days of age are considered to be removed (Fig. 1, Bullet B). This then will remove the first month of the “per product label” 90-day macrocyclic lactone susceptibility gap. If a dog starts regular monthly prevention at the time of diagnosis, then what is left are worms that are between 60 and 120 days of age.

If one accepts that there is 100% efficacy against heartworms less than 2-months of age, one can then consider what happens if dogs are started on regular monthly prevention when heartworms are greater than 2 months of age. It has been reported that there is quite high efficacy against 3-month-old heartworms and fairly good efficacy against 4-month-old heartworms when dogs are treated with macrocyclic lactones. When monthly administration begins against 3-month-old heartworms, the efficacy of the continued monthly treatment of dogs with selamectin (Revolution®, Zoetis), ivermectin/pyrantel (Heartgard®, Merial, now Boehringer Ingelheim), and milbemycin oxime (Interceptor®, Novartis, now Elanco) results in efficacies of 98.5%, 97.7%, and 95.1%, respectively [22] (Fig. 1, Bullet C). At 4 months after infection, the efficacies of Heartgard® and Interceptor® are 96.6% and 41.4%, respectively, and the efficacy of ProHeart® 6 given to dogs with 4-month-old infections is 85.7%, increasing to 97.2% if a second ProHeart® 6 treatment is given again 6 months later [22]. There is, admittedly, still a gap with continuous monthly macrocyclic lactone alone, but it is brief, and only a small percentage of heartworms would continue to mature (Fig. 1, Bullet D). Thus, a bit of a “susceptibility” gap remains relative to any heartworms that may have entered the dog near the time of diagnosis, however, it can be narrowed or closed with the concurrent melarsomine dihydrochloride treatment.

The AHS Guidelines currently state that “melarsomine had not been shown to have activity against heartworms less than 4 months old; recent unpublished data, however, suggest that melarsomine may have more efficacy against juvenile heartworms than previously believed.” Early data showed that melarsomine dihydrochloride was efficacious against 4-month-old worms, and probably more efficacious against these worms than older adults. In one of the first papers dealing with melarsomine dihydrochloride (RM 340), it was stated that “RM 340 is fully effective on *D. immitis* adults (even on young ones of 7 months old) and L5 immatures (4 months old)” [2]. The paper went on to state that a single injection of 2.5 mg/kg melarsomine dihydrochloride had an efficacy of 82.1% against 4-month-old heartworms (all dogs did, however, develop infections with some mature worms). The efficacy was reduced, however, as the heartworms aged; ie, as opposed to the 82.1% kill of heartworms at 4 months of age, a greater percentage of heartworms treated at 7 or 12 months of age with a single melarsomine dihydrochloride injection reached maturity. Efficacy dropped to 55.5% and 51.7%, respectively, with 90% of treated dogs developing mature heartworm infection [2, 9, 26]. In fact, the publication of the NADA in the Federal Register [27] states that “The drug is indicated for the treatment of stabilized, class 1, 2, and 3 heartworm disease (asymptomatic to mild, moderate, and severe, respectively) caused by immature (4-month-old, stage L5) to mature adult infections of *Dirofilaria immitis* in dogs.” Because heartworms apparently become more refractory to the single melarsomine dihydrochloride injection and to macrocyclic lactones as they mature to ages of greater than 4 months, there is seemingly no good reason to delay the first melarsomine dihydrochloride injection. Actually, if one waits 2 or 3 months, the efficacy of the single injection would likely fall closer to the 50% seen in the 7- to 12-month-old worms. Therefore, if a dog begins macrocyclic lactone therapy at the time of diagnosis and receives melarsomine therapy as soon thereafter as is clinically appropriate, a single melarsomine dihydrochloride treatment would be expected to kill 82.1% or so of <4-month-old heartworms. This kill efficacy would fall as the treatment is delayed, per the AHS Guidelines (Fig. 1, Bullet E).

There have been other studies of melarsomine against 4-month-old infections that also suggest good efficacy. In 1989, a study examined several different doses of melarsomine dihydrochloride against 4-month-old heartworms and transplanted adults [28]. Thirty dogs were treated with either 0, 1.6, 1.9, 2.2, or 2.5 mg/kg melarsomine dihydrochloride with two IM injections given 3 h apart. The efficacies were 88.7%, 97.2%, 95.8%, and 100% for the increasing melarsomine levels, with the number of dogs being cleared being 0/6, 2/6, 2/6, and 6/6, respectively. Treating 4-month-old infections with the currently recommended dosage (2.5 mg/kg) twice with the second treatment being given 6 h after the first, the efficacy was 100% (no worms recovered) in one replicate and 95.2% (an average of 1.6 worms in each of four of the five dogs) in the second replicate [28]. This same treatment protocol was used in dogs that received adult heartworms that were 7 months of age; in one group of three dogs the efficacy was 100%, and in a second group of six dogs the efficacy was 97.3% with three of the six control dogs still having an average of 0.5 female worm each [28]. Thus, with this accelerated dosage regimen where the drug was given twice in 3 or 6 h rather than after 24 h, the efficacy was close to 100% against 4-month-old worms (Fig. 1, Bullet E).

Albeit with two IM injections of 2.2 mg/kg given 3 h apart, there is also strong evidence that IM injections of melarsomine dihydrochloride have good efficacy against 4-month-old heartworms based upon a field study [29]. When melarsomine dihydrochloride was first being developed for use in heartworm disease, it was considered as a potential injection that would be given to dogs every 4 months to kill heartworms in the field. For this it is important to understand that melarsomine dihydrochloride is rapidly eliminated from the body in the feces (t_½_ ≈ 3 h; mean retention time ≈ 7 h) [2] with no bioaccumulation [30]. Thus, the plan was to inject dogs every 4 months to kill incoming heartworms. This work was first presented in 1990 at the annual meeting of the American Association of Veterinary Parasitologists where the stated rationale was: “In view of the high level of activity of RM 340 against adult and 4-month-old immatures of *Dirofilaria immitis*, three similar field studies were conducted in states with moderate (GA, FL) or high (LA) enzootic potential to determine the effectiveness of RM 340.” [29, 31] At each study site, 30 naive beagles, allocated to groups of five each, were placed under field conditions for various intervals from April 1988 to April 1989. One group was exposed to infection for 12 months and treated on three occasions, unrelated to mosquito season. A second group was exposed for 8 months and treated on two occasions, during and after mosquito season. These treatments were initiated in August 1988 and consisted of two deep IM lumbar injections of 2.2 mg/kg 3 h apart every 4 months. Heartworms were recovered from all but one of the controls (6/6 in GA ($\overline{x}$ = 6.75; range, 5–8); 6/6 control dogs in FL ($\overline{x}$ = 5.4; range, 1–13); and 4/5 in LA ($\overline{x}$ = 25.2; range, 0–-45). All but one (one worm in one dog in GA) of 15 treated dogs were free of heartworms.

There are no reports of efficacy relative to a single melarsomine dihydrochloride treatment injection against heartworms younger than 4 months of age. The efficacy of the older arsenical thiacetarsamide sodium was found to be most efficacious against 2-month-old worms when compared with 4-, 6-, and 12-month-old worms; the efficacy against 2-month-old heartworms was 99.2% when given at a dose of 2.2 mg/kg BID for 2 days [32]. The efficacy of melarsomine dihydrochloride on 2-month-old worms was 100% [3]. Ten dogs were each infected with 50 heartworm larvae from mosquitoes. Two months after larval inoculation, five dogs were treated with two IM injections of melarsomine (2.5 mg/kg per injection) 24 h apart; and five dogs served as untreated controls. The untreated dogs all developed patent infections. Dogs were euthanatized 7 months after infection, and there were no worms recovered from the treated dogs, while the control group had a mean of 34.6 (range, 24–41) heartworms. Thus, the data suggests that a single melarsomine injection would have efficacy against younger worms (Fig. 1, Bullet F).

**Fig. 2:**
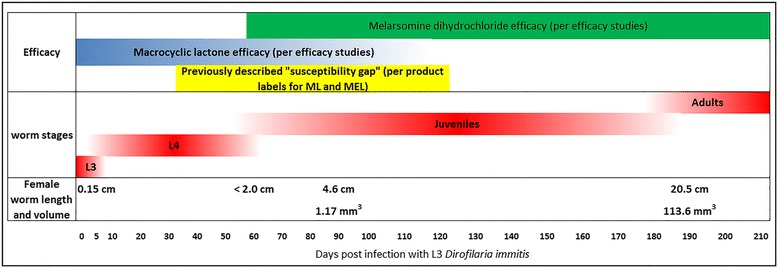
*Dirofilaria immitis* development timeline demonstrationg worm growth over time and the publisehd efficacy of macrocyclic lactones and melarsomine dihydrochloride which, together, support treatment of heartworms at the time of diagnosis, without further delay, as long as the dog is stable and otherwise a good candidate for melarsomine dihydrochloride therapy

The data show that heartworms become more refractory to both macrocyclic lactones and melarsomine dihydrochloride as they mature. If macrocyclic lactone therapy were begun at the same time as melarsomine dihydrochloride and continued for 12 months, it would be expected to kill 95% to 98% of heartworms less than 3 months of age, and depending on the product chosen, 41.4% to 97.2% of any heartworms 4 months of age or younger. A single melarsomine dihydrochloride injection would have efficacy against 2-month-old heartworms (Fig. 1, Bullet F), and then a month later -the two treatments a day apart would have efficacy against both 3-month-old and 4-month-old heartworms. Thus, it would appear that treatment with both melarsomine dihydrochloride and macrocyclic lactone at the time of diagnosis regardless of the duration of infection (days to years) would be optimal for maximal and prompt heartworm clearance. Immature heartworms, 90 days of age at the time of diagnosis, would be 4 months (120 days) old when the second and third injections of melarsomine dihydrochloride are administered (Fig. 1, Bullet E). With the continued macrocyclic lactone administration and the two additional injections of melarsomine dihydrochloride 1 month later, there should be excellent removal of young adult and mature heartworms. At the same time, macrocyclic lactones would prevent new infection. Importantly, larval development in immature infections would be prevented, hence worm biomass would not increase exponentially, as occurs with the 2-month delay advocated by the AHS (Fig. 2).

### Class 1 heartworm treatment

For Class 1 heartworm–infected dogs, which are often treated with only two injections of melarsomine dihydrochloride 24 h apart, the reported efficacy of the two injections is 90.8%. The product label states: “Worms that were too young to be killed by the first treatment series, ie, <4 months, may be killed by a second treatment series.” As described above, however, dogs so treated with the simultaneous initiation of macrocyclic lactone therapy (monthly or sustained release) are likely cleared of most of their younger heartworms. Furthermore, because two melarsomine injections are likely to be highly efficacious against 2-month-old heartworms [3], the label verbiage concerning heartworms <4 months of age is called into question.

### The susceptibility gap and resistant isolates

In light of the fact that dogs may now be infected with heartworms less susceptible to macrocyclic lactone preventives [33], it seems that giving melarsomine dihydrochloride at the start of macrocyclic lactone administration to infected dogs would be a logical step to prevent the further development of heartworms that are less susceptible to macrocyclic lactones. The two modes of pharmacologic action against heartworms are likely different for the arsenical and the macrocyclic lactone, and thus, the combined efficacy of melarsomine dihydrochloride (82%) and the macrocyclic lactones (41%–97%) against heartworms 4 months of age and younger should minimize the chances of such heartworms surviving arsenical therapy. Starting dogs on macrocyclic lactone therapy and treating with at least one melarsomine dihydrochloride treatment upon diagnosis appears to be a logical pretreatment regimen prior to the final two dose melarsomine dihydrochloride treatment.

With the development of Trifexis®, Interceptor® Plus, and Sentinel® Spectrum, it was shown that when dogs were given multiple monthly treatments with milbemycin oxime that was efficacious against the resistant MP3 isolate of heartworms >2 months post infection. In the heartworm efficacy studies during the development of these products, <100% efficacy was seen following both one and two doses of each product when treatment was initiated 30 days after inoculation with L3 *D. immitis*. The 100% efficacy, however, was shown following three doses of Trifexis® and following six consecutive doses of Interceptor® Plus or Sentinel® Spectrum, indicating additional efficacy of these products when administered to dogs with larvae ≥90 days old [34–37]. It appears that the multiple treatments are necessary for 100% prevention against some isolates, and these data also suggest that milbemycin given in multiple treatments kills young, maturing adult heartworms. Combining the heartworm killing effects of macrocyclic lactones with melarsomine dihydrochloride’s effects against worms 2 months of age and older seems to best ensure that resistant worms developing in an infected dog are killed before they can develop to a stage that might put other dogs at risk.

## Conclusions

Currently available data suggests dogs diagnosed with adult *D. immitis* infection should be simultaneously started as soon as possible after diagnosis on both macrocyclic lactone heartworm prevention and the three-dose melarsomine dihydrochloride protocol in order to effectively treat existing heartworms of all ages and to prevent new infections. Delaying melarsomine dihydrochloride treatment for 2 to 3 months following diagnosis should not be recommended as it could be detrimental by allowing further heartworm growth and greater pulmonary vascular damage associated with the death of larger heartworms (Fig. 2).
